# Empathic Behavior and Behavioral Synchrony During Video Conversations in Autistic and Non-autistic Adolescents

**DOI:** 10.1007/s10803-025-06903-y

**Published:** 2025-06-14

**Authors:** Ester Zadok, Zohar Ben-Moshe, Ilanit Gordon, Ofer Golan

**Affiliations:** 1 Department of Psychology, Bar-Ilan University, Ramat-Gan, Israel; 2 Gonda Multidisciplinary Brain Research Center, Bar-Ilan University, Ramat-Gan, Israel

**Keywords:** Autism, Behavioral synchrony, Facial expression synchrony, Empathic behavior, Video conversations, Adolescents

## Abstract

Behavioral synchrony is an important component of successful social interaction. Synchronization of facial expressions during interaction is considered a facilitator of empathic behavior. Autistic individuals show alterations in synchronous and empathic behaviors. Yet, an ecological examination of these among adolescents is needed. Through a video-based platform, we employed a social interaction sequence with a nonautistic confederate that included a friendly conversation and an expression of discouragement. 50 male adolescents (23 autistic) participated. Socio-emotional behaviors, assessed by naïve trained raters and by automated measure of facial expressions, were analyzed. Results indicated reduced synchrony in interactions with autistic adolescents, compared to non-autistic adolescents, as well as fewer empathic behaviors. Participants’ emotion recognition abilities did not account for these differences. Our findings point to the central role of behavioral synchrony in the social disparities autistic adolescents often experience.

Behavioral synchrony, the spontaneous coordination and congruence of behaviors between interacting partners in both form and timing (Delaherche et al., 2012), is considered to reflect, as well as to facilitate, rapport and smoothness of the interaction (Chartrand et al., 2006; Shamay-Tsoory et al., 2019). During social interactions, individuals automatically synchronize their tones of voice (Neumann et al., 2000), eye movements (Richardson et al., 2007), facial expressions (Hess et al., 2001), and their postures and gestures (Lakin et al., 2003). In addition, coordination of motion, irrespective of the specific movements, has been found between unacquainted individuals, while conversing (Tschacher et al., 2014) and during shared activities (Gordon et al., 2020).

Through its different modalities, behavioral synchrony has been linked to perspective taking (Chartrand et al., 1999), mentalizing (Baimel et al., 2018) and empathy (Valdesolo et al., 2011). Empathy is a multifaceted construct involving several components. The affective component, often referred to as emotional empathy, pertains to the ability to perceive and be sensitive to the emotional states of others (Decety et al., 2016). The cognitive component, which Dvash and Shamy-Tsoory (2014) conceptualized as Theory of Mind (ToM), involves understanding another’s emotions while maintaining a distinction between their emotions and one’s own. Empathic behavior, often associated with pro-social behavior (Decety et al., 2016), refers to the capacity to respond appropriately to the emotional states of others. Following this multifaceted model, when a conversational partner receives distressing news, the components of empathy might involve perceiving their distress (affective), understanding their emotional state without identifying with it (ToM), and reacting appropriately, such as allowing for a conversational halt, offering support or asking, ‘Is everything alright?’.

The relationship between behavioral synchrony, perspective taking, mentalizing and empathy is bidirectional in nature (Shamay-Tsoory et al., 2019) such that behavioral synchrony both reflects and supports these psychological and cognitive capabilities. As such, findings from experimental studies that manipulated synchrony in dyads showed a causal role of behavioral synchrony in mentalizing and emotional responding in interactions (Baimel et al., 2018; Valdesolo et al., 2011).

Specifically, facial expression synchrony, i.e., the congruent facial reactions to the emotional facial displays of others, plays an important role in socio-emotional communication (Hess et al., 2001). When interacting, the observer automatically imitates the emotional expression of another, and reactivates, to some degree, the implied emotional state in sensory, motor, and affective systems. This simulation supports the inference of meaning of the facial expression, and eventually, of the emotional state of the other (Niedenthal, 2007). As it appears in the early stages of processing a facial expression, such simulation may precede cognitive processes of mentalizing and perspective taking. In an interactive context, an ongoing and reciprocal simulation of the other’s expressed emotion would form facial expression synchrony. Indeed, facial expression synchrony has been suggested to foster an understanding of the emotional meaning, and to facilitate empathy (Decety & Jackson, 2004). Mutual smiles, particularly, were found to predict affiliation of dyad members (Golland et al., 2019) and helping behaviors (Tunçgenç & Cohen, 2018).

Despite the above-mentioned prosocial consequences of behavioral synchrony, there is also accumulating evidence demonstrating that more behavior synchrony is not necessarily always better (Abney et al., 2015; Butler, 2015; Dahan et al., 2016; Feniger-Schaal et al., 2016; Randall et al., 2011). Instead, it has been suggested that flexibly going in and out of synchrony should also be measured and examined as an adaptive indicator of social reciprocity (Mayo et al., 2020). Furthermore, shifts from synchrony to asynchrony or no synchrony may foster empathy, as they strengthen a sense of connectedness with others, while also refining self-other differentiation (Behrends et al., 2012). In other words, for an empathic response to take place, one must recognize that the emotion he or she is experiencing, as part of the emotional simulation and resonance, is the reflection of the other’s emotional state. If the observer is unable to down-regulate his/her own emotions, the emotional situation may become overwhelming (Decety & Lamm, 2009) and thus an empathic behavior is less likely to be expressed. Instead, the distressed observer would seek to alleviate his own arousal (De Waal, 2008). This might be particularly difficult for autistic individuals.

Autism is a neuro-developmental condition, characterized by difficulty in social communication, circumscribed (i.e. intense) interests and rigid and repetitive patterns of behavior (American Psychiatric Association, 2013). Prior research investigated how interpersonal synchrony mediates the relationship between autism, interaction quality and outcomes, revealing diminished or atypical behavioral synchrony (McNaughton & Redacy, 2020) alongside difficulties in self-regulation and altered empathic behaviors. (Mazefsky et al., 2014; Zadok et al., 2023). Whereas past views of empathy deficits in autism have been challenged over the years (Fletcher-Watson & Bird, 2020), revealing intact, and even intense affective empathy in autistic individuals, differences in social understanding still occur, and may lead to an altered empathic, or prosocial behavioral response. Moreover, interactions that include an autistic individual and a non-autistic individual are characterized by atypical behavioral synchrony, compared to interactions of two non-autistic partners, across various behavioral domains. As demonstrated in a review by McNaughton and Redcay (2020), who pointed to persistent findings of reduced motor, conversational, physiological, and neural synchrony in dyads of autistic and non-autistic partners. Furthermore, a meta-analysis by Trevisan et al., (2018) established less facial expression synchrony in autism, in both spontaneous and intentional mimicry. Moreover, computer vision analysis revealed less facial expression coordination among autistic youth during positive and cooperative conversations with a Non-Autistic (NA) partner (Zampella et al., 2020). In a different study, less synchronization of speech features, including volume, pitch, and speech rate, was demonstrated (Ochi et al., 2019). Emphasizing the notion of an adaptive interpersonal synchrony as a flexible one, Mayo and Gordon (2020) have shown that the flexibility in eye gaze synchronization between autistic children and their parents, measured from entries and withdrawals from synchrony, was related to the child’s social functioning, whereas the total duration of synchrony was not.

Another mechanism, more cognitive in nature, that has been suggested to link to empathic behavior, relates to facial emotional recognition, i.e., the perceptual ability to infer emotion from facial expressions (Holland et al., 2021). Traditionally, difficulties in emotion recognition have been considered a well-established feature of autism (Baron‐Cohen et al., 1997; Bölte et al., 2003; Frith & Happé, 1994). Moreover, such difficulties were suggested to relate to atypical socio-emotional behaviors, including empathic behavior (Baron-Cohen et al., 2009) and behavioral synchrony with a social partner (Fitzpatrick et al., 2018). Nevertheless, other findings indicated similar emotion recognition abilities between autistic and non-autistic individuals (Arnaud, 2022; Harms et al., 2010). Thus, the centrality of emotion recognition disparities in the socio-emotional behavior of autistic individuals calls for further investigation.

This study aimed to examine empathic behaviors, behavioral synchrony, and facial expression synchrony of autistic and non-autistic adolescents during conversation with a non-autistic partner. The interactions were based on an ecological paradigm (Pro-social & Empathic Behavioral Assessment (PEBA); Zadok et al., 2021) that included a friendly conversation with a research confederate, a response to her discouragement, and a return to a friendly conversation. In the current study, these interactions took place through a video interaction platform.

Examining video-based social interactions appears to be increasingly relevant to the contemporary social world. The prosperity of online social networks, as well as the social distancing restrictions introduced during the COVID-19 pandemic, have made video-based interactions a common method for socializing (Hamilton et al., 2022). Moreover, studies with non-autistic individuals suggest an emergence of empathy in video-based interactions, during remote psychotherapy sessions (Sperandeo et al., 2021) and experimental tasks (Nguyen, & Canny, 2009). Nevertheless, research on the use of video-based interactions with autistic individuals has generally focused on diagnostic assessments (Boisvert et al., 2010) and interventions (Ferguson et al., 2019) and aimed to establish their feasibility. Notably, video conferencing platforms commonly experience transmission delays, with potential lags of up to 200 ms (Chang et al., 2021). These delays may pose challenges in precisely gauging synchrony during video interactions and may also influence participants’ subjective perception of synchrony. We aimed to overcome these limitations by comparing the online behavior of autistic adolescents to that of non-autistic adolescents, who may experience similar delays. To our knowledge, the current study is the first to focus on social behavior, particularly synchronous and empathic behavior, during video-based interaction of autistic individuals with non-autistic partners. Moreover, despite the increasing use of video-based interaction among adolescents (Hamilton et al., 2022), these important aspects of behavior were not examined in this age group, whether in autistic or NA development.

To that purpose, we used naïve coders assessments of behaviors during the social interaction, as well as an automated measure of facial expression analysis. Considering the friendly and primarily positive context of the interaction, as well as the central role of smiles in forming connectedness (Golland et al., 2019), the automated measure focused on synchrony of joyful facial expressions. In addition, this study included an emotion recognition task, to examine relationships between prior mentalizing abilities and observed behaviors during the social interaction.

Considering previous studies, we hypothesized that interactions with an autistic adolescent would be characterized by reduced behavioral synchrony, including facial expression synchrony, compared to interactions with NA adolescents. The latter were expected to display shifts in synchrony, specifically, a reduction in synchrony when facing discouragement, whereas interactions with autistic adolescents were expected to be more constant in their synchrony dynamics. Furthermore, we hypothesized that autistic adolescents would display less empathic behavior in response to the interaction partner’s discouragement, compared to non-autistic adolescents. Finally, the relationship between emotion recognition alterations and synchronous and empathic behaviors during the interaction was examined in an exploratory manner.

## Method

### Participants

Fifty male adolescents, out of whom 23 were autistic, aged 13–18 years, took part in the study. Participants of both groups were invited to take part in a social interaction study, through advertisements in the community and social networks. The participants were native Hebrew speakers, with medium–high socio-economic status (*M* = 6.51, *SD* = 2.12), based on the ranking of their residential place (Central Bureau of Statistics, 2023). The ranking system allocates each municipality to a SES cluster on a 1–10 scale, with higher clusters indicating higher SES. The Social Responsiveness Scale (SRS-2; Constantino et al., 2012) was used to screen-out non-autistic participants who exceed the cut-off for suspected autism. One non-autistic participant was excluded for this reason. Inclusion criteria for the autism group was a formal diagnosis of ASD with no intellectual impairment given by a clinical psychologist or a psychiatric, and a recognition of the diagnosis by the Israel Ministry of Education and the National Insurance service. Additionally, parent report on the SRS-2 was used to support the existing diagnosis, according to a cutoff of a T score of 60. All the participants completed the matrix and vocabulary subtests from the Hebrew versions of the WISC-IV (Wechsler, 2003) or the WAIS-III (Wechsler, 1997), according to their age. The groups were comparable on age and Wechsler scores. Table 1 presents averages and standard deviations of participant demographics of the final sample (*N* = 49). The study was ethically approved by the Institutional Review Board of Bar-Ilan University.

**Table 1:** Demographic data of the autistic and non autistic groups

	Autistic N = 23	NA N = 26	t (47)
	M(SD)	M(SD)	
Age	15.54(1.77)	15.59(1.54)	− 0.10
Wechsler vocabulary	10.74(2.89)	11.65(1.81)	− 1.34
Wechsler matrix reasoning	9.17(3.71)	10.31(3.21)	− 1.15
SRS-2	71.59(9.47)	41.77(4.06)	13.74***

Note: SRS-2—social responsiveness scale 2nd edition; ***p < .001

### Measures

#### Pro-social & Empathic Behavioral Assessment (PEBA; Zadok et al., 2021)

This measure is based on observations of a semi-scripted interaction sequence and a corresponding global behavioral coding system (detailed below) which examines the participant’s empathic and pro-social behavior in response to a friendly stranger’s discouragement. Following 3 min of a friendly conversation, during which the participant and the confederate are asked to get to know each other, the confederate expresses discouragement for a minute, and then resumes a friendly conversation for another minute. Compared to previous administration of the paradigm (Zadok et al., 2021), the administration of the measure in the current study included two modifications: First, instead of receiving a text message informing the confederate she has failed an exam (which served as the trigger for the discouragement stage), the confederate referred to such a failure as part of the ongoing conversation. Meaning, after 3 min of a friendly conversation, the confederate changed the conversation topic to talk about school and studies and shared with the participant that she is failing. While doing so, she demonstrated discouragement verbally (“I’m really struggling with my studies, I just got a pretty bad grade”) and nonverbally (slowing of nonverbal communication). By that, we aimed to examine whether refined socio-emotional cues yield discrete responses in empathic behavior among autistic and NA adolescents. Second, the interactions took place through the Zoom video conversation platform. In the Zoom meeting, participants were asked to use “speaker view”, which enabled them to watch the confederate in a central, large frame, and themselves in a smaller, marginal frame.

The confederates were two female undergraduate students, trained according to the PEBA administration protocol by (see Zadok et al., 2021). To maintain fidelity to the amended protocol, they were provided with corrective feedback routinely throughout the trial. The confederates were naïve to participants’ diagnosis, and to the research hypotheses. They were randomly assigned to the interactions according to their availability. There was no significant association between the confederate assigned and the participant’s diagnosis (χ^2^(1) = 1.27, *p* = 0.26).

The interaction was recorded (using the Zoom recording function) and its last 3 min—comprising three interaction stages: friendly, discouragement, and return to friendly stages—were analyzed. To ensure that analyses are not performed on portions of the interactions that may be impacted by technical issues (such as freezing or lagging), the recordings were examined by the authors and such video portions were removed. The interactions were analyzed in two ways: global behavioral coding, and using the iMotions Affectiva automated engine.

#### Global Behavioral Coding

Each of the three interaction stages was globally coded on 5-point Likert scales (higher scores indicated a greater level of the behavior) by trained raters: two undergraduate psychology students trained according to the PEBA coding protocol by the first author (see Zadok et al., 2021). The raters were naïve to participant’s diagnosis, and to the research hypotheses. Synchronization of eye gaze and synchronization of body posture (which were included in the original coding system) were not coded in this study, due to the use of video conversations which limited our observations to the upper bodies of the interaction partner, and hindered assessment of eye gaze directions. Following training, and before proceeding to coding study videos, interrater reliability was based on the coding of six training videos, by calculating agreement ratios between each coder and the first author. Following Ratto et al., (2011), agreement was counted when similar codes were given to specific item, within one coding interval. The following coded behaviors were averaged and grouped into two domains. First, *Behavioral Synchrony*—synchronization of three nonverbal characteristics of behavior to that of the social partner: 1. Vocalizations; 2. Facial expression; 3. Affect. Each item was coded on a Likert scale of 1 to 5 (e.g., 1 = clearly desynchronized, 3 = somewhat synchronized throughout the coded time section or well synchronized only in a small part of the coded time section, 5 = well synchronized for most of the coded time section). The second domain was *Empathic Behaviors*: comments, interest, prosocial behavior, and social understanding. Likewise, each item was coded on a Likert scale of 1 to 5 (e.g., comments: 1 = no comments or irrelevant comments, 3 = on topic but inappropriate comments, 5 = appropriate and relevant comments; prosocial response: 1 = no prosocial response, 3 = expressions of concern, however hesitated, awkward, or exaggerated; 5 = appropriate expression of care towards the confederate). Empathic behaviors were coded only for the discouragement and return to friendly stages, due to the change of the conversation context in those stages.

To maintain reliability, 20% of coded interaction videos collected were co-coded by the first author. In cases of disagreement the third author was consulted, and raters were offered corrective feedback. Intraclass correlation coefficients (ICC) were calculated to assess inter rater reliability of each coded behavior. Average ICCs (3,1) for a two-way mixed effects model (Shrout & Fleiss, 1979) were as follows: vocalization (0.50), facial expression (0.73), affect (0.72), comments (0.68), interest (0.95), prosocial behavior (0.80), social understanding (0.57). Internal consistency for the *Behavioral synchrony* domain was 0.75 for the friendly stage, 0.69 for the discouragement stage, and 0.74 for the return to friendly stage; for the *Empathic Behavior* domain internal consistency was 0.87 for the discouragement stage and 0.75 for the return to friendly stage.

#### iMotions Affectiva (iMotions, 2015)

Video recordings were imported into iMotions Biometric Research Platform and analysed using the Affectiva AFFDEX engine. Building on Friesen and Ekman emotion action unit mapping (Ekman & Friesen, 1969), the engine calculates the likelihood of an emotion out of observed facial expressions and action units. Joy, which we focused on, was considered more likely in the presence of smiles, and in the absence of brow raises and brow furrows.

The Affectiva facial expression recognition software was validated vis-a-vis EMG measurements (Kulke et al., 2020). No significant differences were found between Affectiva’s detection of happy facial expressions in autistic children and in their NA peers (Banire et al., 2020).

**Fig. 1:**
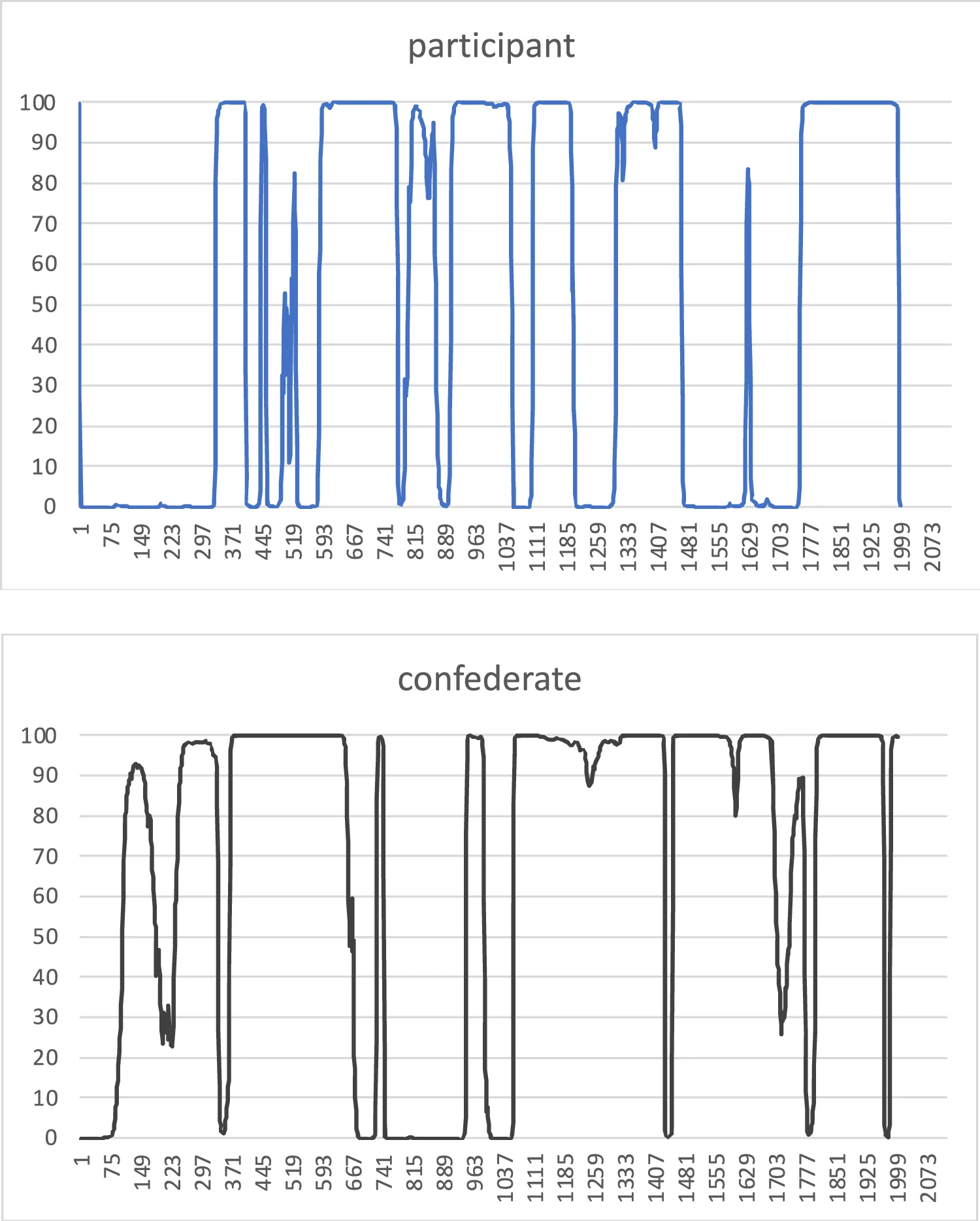
An example of facial expression data of one interactive pair in the friendly stage: Y-axis = probabilities of a joyful facial expression, X-axis = frames

For each face, the analysis provided probabilities that a joyful facial expression was expressed during the interaction, in a sampling rate of 30 Hz. Thus, for each dyad we extracted two temporally parallel series of joy expression probabilities, one of the participant’s and one of the confederate’s. Data was analyzed as follows: First, each dyad’s data series were examined for missing data, meaning, frames in which the faces of the participant, confederate or both were not recognized. Such frames were removed from the data and the proportion of frames deleted in each interaction was calculated. Next, to exclude extreme cases of unusable data, an outlier analysis was conducted, revealing four participants—three in the autistic group and one in the NA group—with extreme values of unusable data on any of the three stages (53% and 60% on the discouragement stage, 68% 78% and 81% on the return to friendly stage).Therefore, these four participants were excluded from the automated facial expression analysis. Then, in order to examine if the proportion of unusable frames is the result of arbitrary technical issues or if it is related to participants’ behavior, a repeated measures MANOVA was conducted on the proportion of unusable data, with stage as a within subject factor and group as the between group factor. The analysis revealed a significant main effect for group (*F*(1,40) = 4.41, *p* = 0.04, *η*^2^ *p* = 0.1) indicating higher percentage of missing data for autistic (*M* = 0.20, *SE* = 0.03) compared to non-autistic (*M* = 0.10, *SE* = 0.02) participants. In addition, a main effect for stage was significant (*F*(2,39) = 3.55, *p* = 0.04, *η*^2^ *p* = 0.15). Post-hoc analysis indicated the percentage of missing data in the return to friendly stage (*M* = 0.17, *SE* = 0.02) was significantly higher than in the discouragement stage (*M* = 0.12, *SE* = 0.02; Mean difference *i − j* = 0.06, *SE* = 0.02, *p* = 0.03), whereas the percentage of missing data in the friendly stage (*M* = 0.14, *SE* = 0.02) did not differ from that of the other two stages. The group by stage interaction was not significant (*F*(2,40) = 4.23, *p* = 0.05, *η*^2^ *p* = 0.1). These findings suggest the missing data indeed relates to participants’ behavior. Next, to compute facial expression synchrony in the dyads, the rMEA R package (Kleinbub & Ramseyer, 2022) was used. Originally aimed to quantify motion synchrony, the rMEA was used to perform cross-correlation functions for positive and negative time-lags in a time window of 5 s, by shifting the data one frame at a time (i.e., in steps of 0.03 s). Then, results were standardized (Fisher’s Z) and averaged. Finally, for each dyad at each of the paradigm’s three stages (i.e., friendly, discouragement and return to friendly) one value was extracted, which represented joy expression synchrony averaged across all possible lags. For visualization of facial expression synchrony data, see Fig. 1.

#### Emotion Recognition Task

**Fig. 2:**
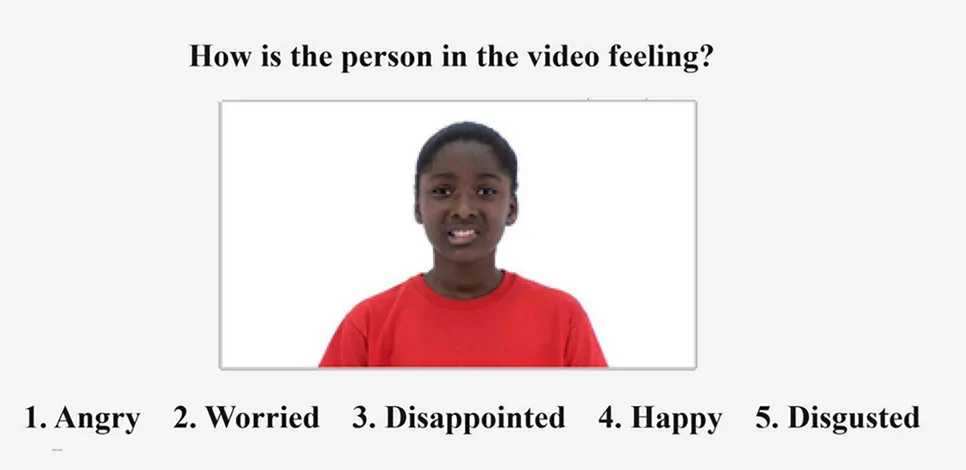
An example of an item included in the emotion recognition task

Based on the EU-Emotion Stimulus Set (O’Reilly et al., 2016), this task aimed to assess emotion recognition abilities. This set includes dynamic stimuli of emotions and mental states represented through different expression modalities, including short videos of facial expressions, by child and adult actors from different ethnical groups. The set was cross-culturally validated in several countries, including Israel (for further information regarding the stimulus set and its validity, see O’Reilly et al., 2016). For this study, 12 complex emotions were represented by 3 videos each. We decided to include only complex emotions and mental states because previous studies showed that autistic individuals with intact intellectual functioning recognize simple emotions correctly (Golan et al., 2006). Thus, tasks that focus on basic emotions may miss more subtle social cognition variations. To validate the set of items we chose, it was completed by 24 non-autistic adolescents, recruited through ads in social networks. Items with success rate lower than 42.9% were discarded, according to binomial probability distribution with significance level of 0.05. In addition, items including distractors that were mistakenly recognized as the target emotion by more than 40% of the participants were discarded. Finally, 24 valid items comprised the stimuli set, representing 8 complex emotions: disappointment, shame, worry, hurt, frustration, playfulness, pride, and interest. For each item, the video was automatically played, and at its end, the frame froze. During that display, five emotions and mental state labels appeared on the screen, and the participants were guided to determine which emotion was expressed. A screenshot illustrating an item included in the task is presented in Fig. 2. Kuder-Richardson-20 (KR-20) Reliability analysis indicated good internal consistency (*α* = 0.80) for the task.

#### Social Responsiveness Scale, 2nd edition (SRS-2; Constantino & Gruber, 2012)

The SRS-2 is a 65-item rating scale measuring the level of autism features as they occur in natural social settings. Completed by parents, the SRS-2 is appropriate for use with children from 4 years of age to adulthood. Subscales include social awareness, social cognition, social communication, social motivation and mannerisms. Internal consistency for SRS-2 subscales was reported as 0.91–0.97, with test–retest reliability of 0.84–0.97 and interrater reliability of 0.74–0.95 (Bolte et al., 2008). In the current study, parents completed the SRS-2 online.

### Procedure

Participants were invited to a one-hour online experiment session. Following assent and parent consent (obtained through digitally signed forms), participants completed the Wechsler matrix and vocabulary subtests in an initial Zoom meeting with an experimenter. Next, they joined a different Zoom meeting in which they met a confederate and took part in the PEBA interaction sequence. Finally, they were asked to complete the emotion recognition task via Qualtrics, with an experimenter available for questions on the phone or via WhatsApp.

### Statistical Analyses

Statistical analyses were performed in R statistical software (version 3.6.2) and Jamovi (version 1.6.23). Since the PEBA domain scores (i.e., empathic behavior and behavioral synchrony) were negatively skewed, group differences throughout the interaction stages were examined by the ANOVA-type test (Brunner et al., 1997), a nonparametric analysis (ranked-based) of longitudinal data in factorial settings using the nparLD R package (Noguchi et al., 2012). The F1-LD-F1 design was chosen, with group as a whole-plot factor (i.e., a between groups factor) and interaction stage as a sub-plot factor (i.e., a repeated measure). Two analyses were performed, with behavioral synchrony scores and empathic behavior scores as dependent variables, generating a 2X3 and a 2X2 designs, respectively. ANOVA-type statistics and their significance were examined.

Next, to validate the facial expression synchrony measure as representing meaningful coordinated expressions, we compared the actual data with shuffled data generated by the rMEA package, which reflects coincidental synchrony between conversational partners that did not actually interact- with each other. To that end, 3000 possible combinations were randomly selected, and their averaged synchrony correlation was computed and compared to each group’s mean synchrony correlation. Finally, group differences in joy synchrony correlations throughout the interaction stages were examined by a mixed design two-way ANOVA (2 × 3), with group and stage as factors.

## Results

First, differences between confederates were checked for all coded behavioral scores and *Affectiva* automatically yielded scores. No confederate differences were found (P > 0.1 for all).

Group differences were tested for coded behavior and for automatically measured facial expression synchrony. Group main effects and Group by stage interaction effects were examined, using Holm’s sequential rejective Bonferroni procedure (Holm, 1979) as correction for familywise type I error rates. Post-hoc achieved power was computed and found to be 0.7.

### Coded Behavior

**Fig. 3:**
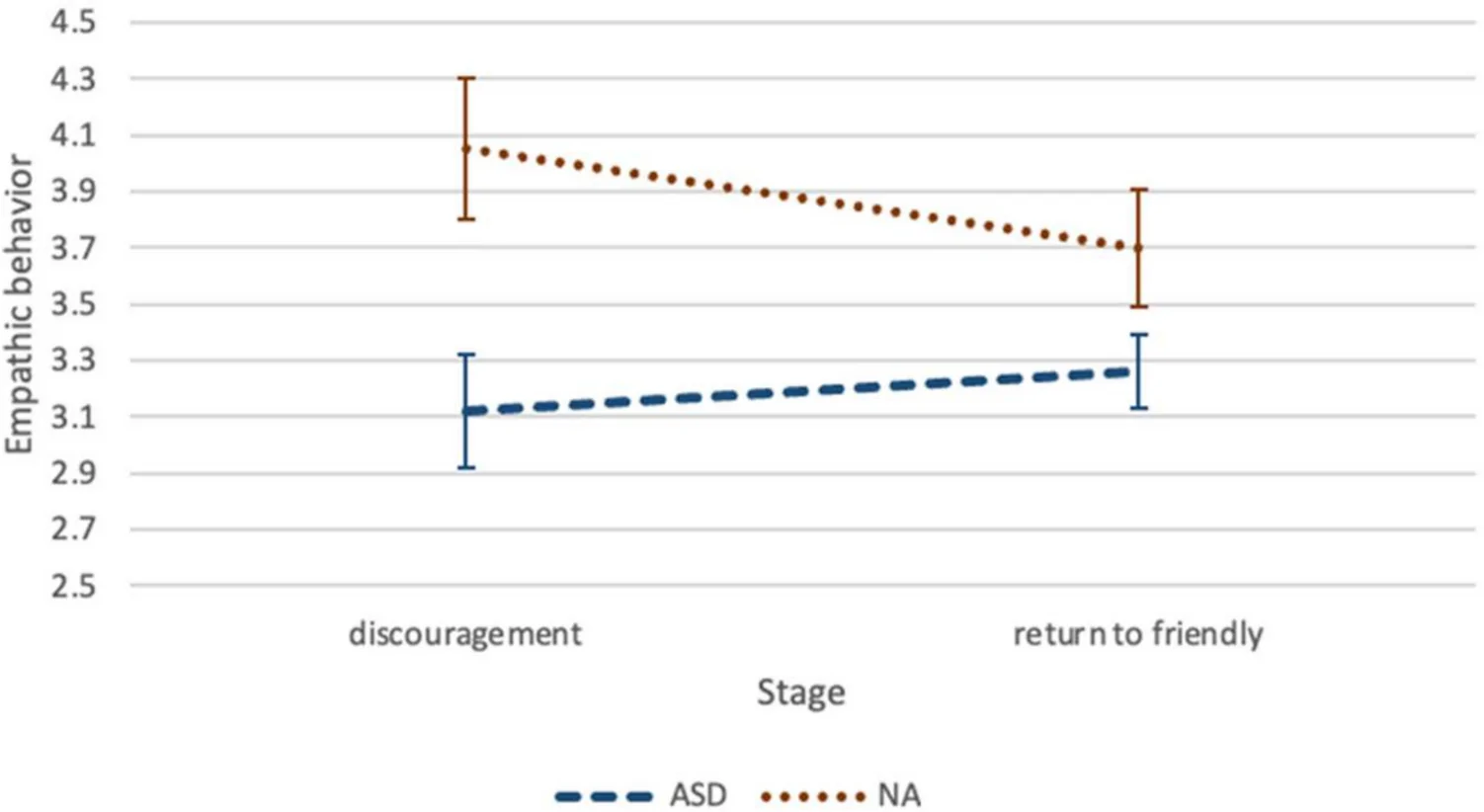
Mean scores of empathic behavior (with standard error bars) of the autistic and NA groups

Group differences in coded behavior, i.e., the PEBA domain scores of empathic behavior and behavioral synchrony, were examined by a nonparametric analysis. A first ANOVA-type test examining empathic behavior revealed a significant main effect for group (*ATS* = 5.67, *p* = 0.01, *η*^2^*p* = 0.12), indicating lower empathic behavior scores among autistic adolescents (rank mean = 40.39) compared to non-autistic adolescents (rank mean = 57.56). The interaction effect (stage X group), after a Holmes Sequential Bonferroni correction, was marginally significant (*ATS* = 6.04, *p* = 0.01, *η*^2^_*p*_ = 0.10). Pairwise comparisons indicated that whereas among autistic adolescents’ empathic behavior levels did not change from the discouragement stage (rank mean = 39) to the return to friendly stage (rank mean = 41.78; *W* = *93.5*, *p* = 0.77), among non-autistic adolescents empathic behavior levels were higher in the discouragement stage (rank mean = 63.54) compared to the return to friendly stage (rank mean = 51.58; *W* = 224, *p* = 0.0013). See Fig. 3.

**Fig. 4:**
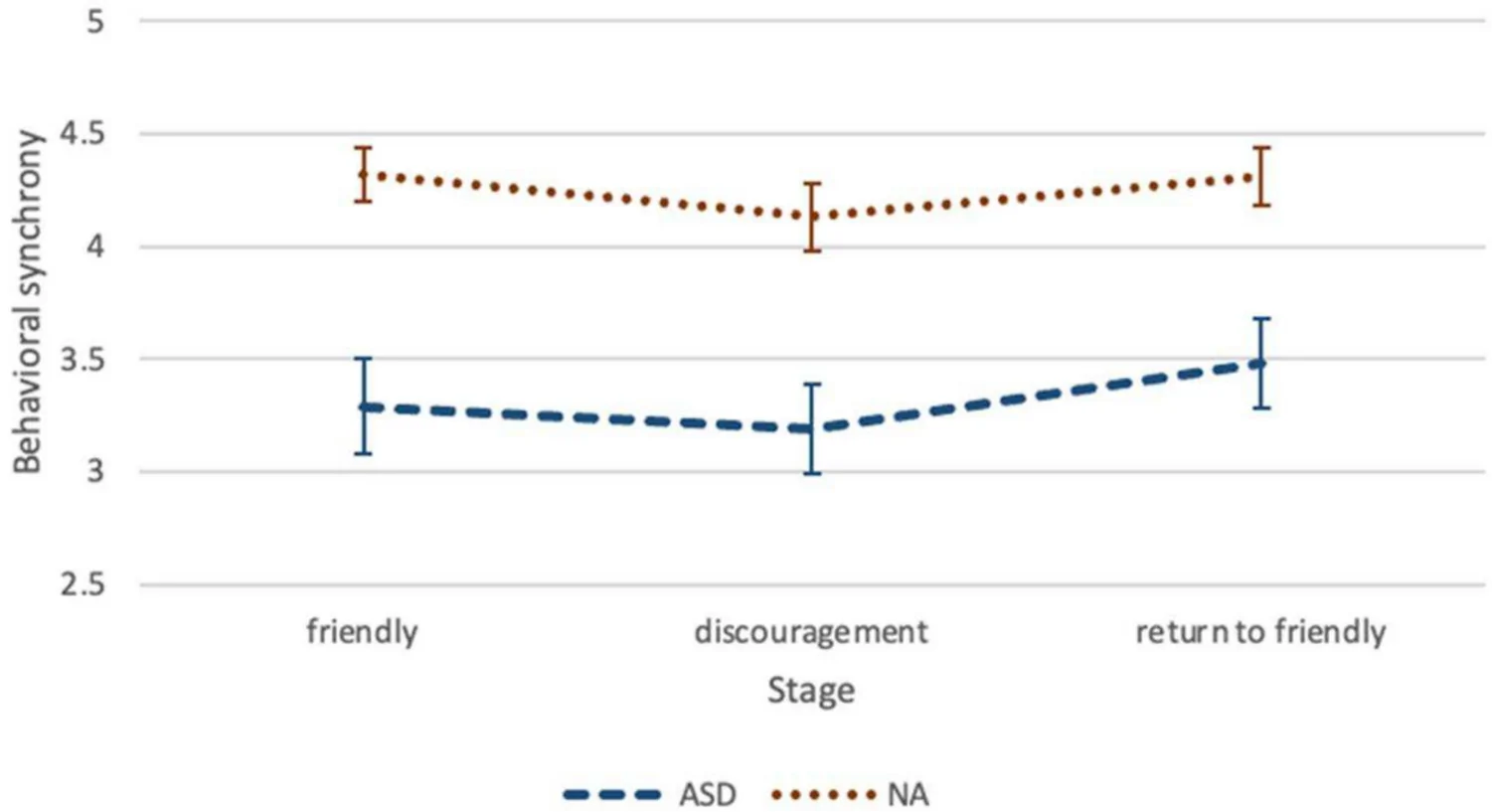
Mean scores of behavioral synchrony (with standard error bars) of the autistic and NA groups

A second ANOVA-type test examining behavioral synchrony revealed a significant main effect for group (*ATS* = 15.24, *p* < 0.001, *η*^2^_*p*_ = 0.29) indicating lower behavioral synchrony scores among autistic adolescents (rank mean = 53.54) compared to non-autistic adolescents (rank mean = 92.10). The interaction effect (*ATS* = 0.28, *p* = 0.80) was not significant. For illustration of the groups mean scores in each interaction stage, see Fig. 4.

### Facial Expression Analysis

Preliminary t-tests comparing our data with shuffled data (which had a mean correlation of − 0.002) revealed that the real joy synchrony correlations were significantly higher than the shuffled data both in dyads with autistic adolescents (*t*(18) = 2.21, *p* = 0.04, *d* = 0.51) and in dyads with non-autistic adolescents (*t*(23) = 7.55, *p* < 0.001, *d* = 1.54).

**Fig. 5:**
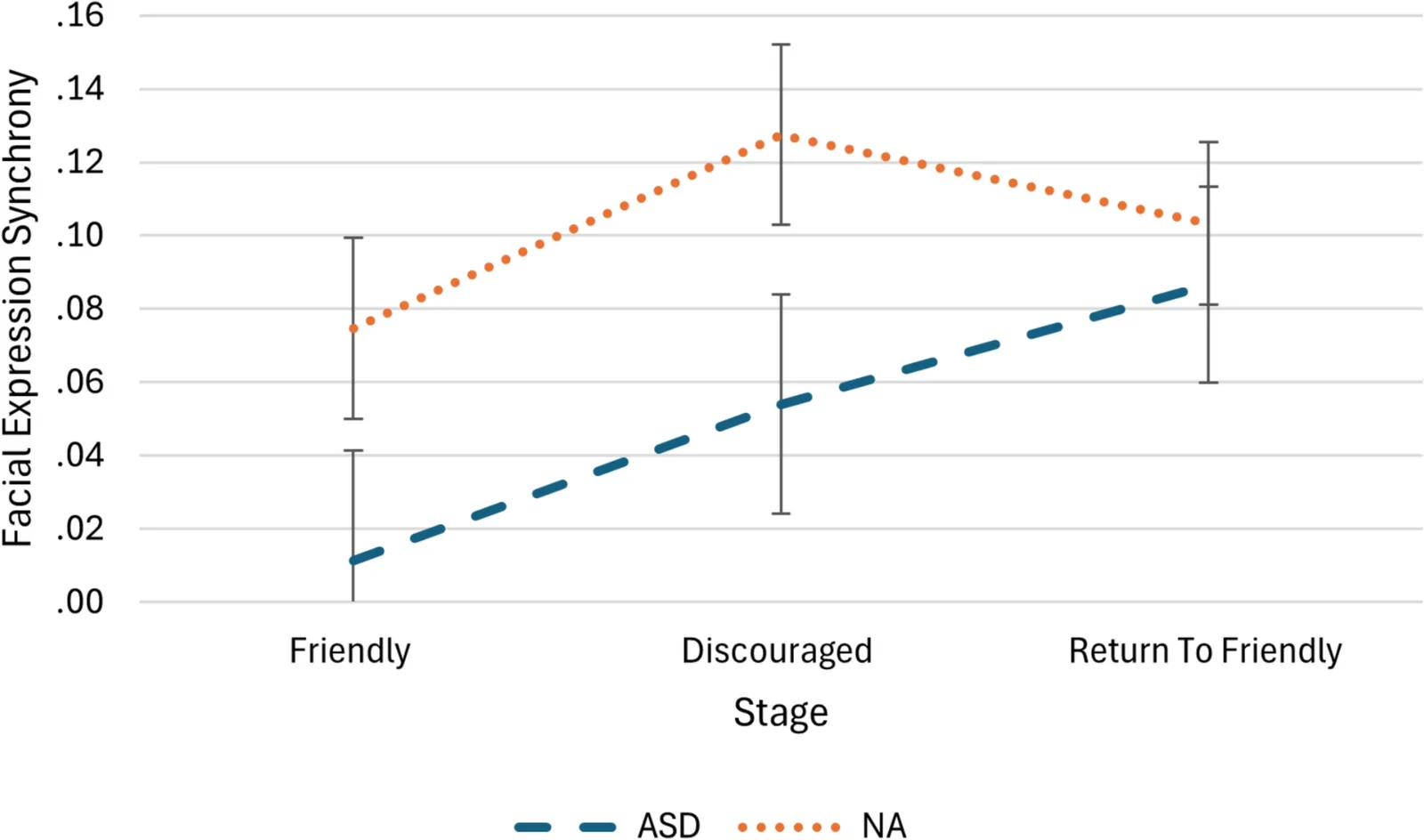
Facial expression synchrony means (with standard error bars) of the autistic and NA groups

Next, a MANOVA for repeated measures examining group differences throughout the interaction stages was conducted. Due to group differences in the proportions of missing data, average proportion of missing data was entered as a covariate. Results revealed a marginally significant main effect for group (*F*(1,39) = 3.21, *p* = 0.08, *η*^2^_*p*_ = 0.08), indicating more synchrony in dyads with non-autistic adolescents (*M* = 0.10, *SE* = 0.02) compared to dyads with an autistic adolescent (*M* = 0.05, *SE* = 0.02). The group by stage interaction was not significant (*F*(2,38 = 0.5, *p* = 0.48, *η*^2^_*p*_ = 0.01). The effect of the covariate was also non-significant (*F*(2,38) = 0.86, *p* = 0.43, *η*^2^_*p*_ = 0.04). Furthermore, the covariate by stage interaction was non-significant (*F*(2,38) = 0.25, *p* = 0.78, *η*^2^_*p*_ = 0.01). Facial expression synchrony means in each group are presented in Fig. 5.

### Emotion Recognition Scores

Group differences in emotion recognition task scores were examined by a t-test, indicating higher scores among non-autistic adolescents (*M* = 15.96, *SD* = 2.32) than among autistic adolescents (*M* = 14.26, *SD* = 3.16; *t*(47) = − 2.16, *p* = 0.03, *d* = − 0.62). Additionally, correlations between the task scores and coded behavior (i.e., behavioral synchrony scores and empathic behavior scores) and facial expression synchrony were examined. The task scores did not correlate significantly with any of the above, either in the autistic group only, or in the full sample. The correlations are presented in Table 2.

**Table 2:** Spearman correlations (*p* = *n.s.*) between the emotion recognition task scores and coded behavior (i.e., behavioral synchrony scores and empathic behavior scores) and facial expression synchrony, across the interaction stages: friendly, distress and return to friendly stages

	Empathic behavior		Behavioral synchrony			Facial expression synchrony		
	Distress	Return to friendly	Friendly	Distress	Return to friendly	Friendly	Distress	Return to friendly
Emotion recognition task scores of the autistic group	.00	.07	.08	.03	− .01	− .08	.16	.03
Emotion recognition task scores of the full sample	.19	.15	.15	.11	.02	.05	− .06	− .18

### Study Measures and SRS-2 Scores

We explored the correlations between the SRS-2 scores of both autistic and non-autistic participants, and their empathic behavior, behavioral synchrony, and facial expression synchrony scores. After a correlation correction, SRS-2 scores had a significant correlation with behavioral synchrony in the friendly stage (*r*_*s*_ (49) = − 0.61, *p* < 0.001), discouragement stage (*r*_*s*_ (49) = − 0.45, *p* = 0.001) and return to friendly stage (*r*_*s*_ (49) = − 0.49, *p* < 0.001). Furthermore, SRS-2 scores had marginally significant correlations with empathic behavior in the discouragement stage (*r*_*s*_ (49) = − 0.36, *p* = 0.01), and in the return to friendly stage (*r*_*s*_ (49) = − 0.32, *p* = 0.03). Additionally, SRS-2 scores had a significant correlation with facial expressions synchrony in the friendly stage *(r*_*s*_ (41) = − 0.45, *p* = 0.004). However, SRS-2 scores were not significantly correlated with emotion recognition (*r*_*s*_ (49) = − 0.09, *p* = 0.54).

## Discussion

This study aimed to investigate behavioral synchrony and empathic behavior among autistic and non-autistic adolescents, while they interact with a friendly, but also discouraged, stranger, on a video-based conversation. Our findings demonstrated that throughout the interaction, autistic adolescents displayed lower levels of empathic behavior and behavioral synchrony, as indicated by assessments of naïve coders. Moreover, among non-autistic adolescents, empathic behavior was higher in the discouragement stage than in the return to friendly stage, whereas among autistic adolescent such a change was not observed. Automated facial expression analysis also revealed a trend of lowered synchrony in dyads with a NA confederate and an autistic adolescent compared to dyads with a non-autistic confederate and a NA adolescent throughout the interaction.

As hypothesized, autistic adolescents showed less empathic behaviors towards the social partner, when she shared her discouragement and when she restored a friendly conversation. This behavior appeared more constant than non-autistic adolescents’ empathic behavior, as only the latter’s was higher in the face of the partner’s discouragement than when friendly conversation resumed. This is in line with previous literature indicating difficulties in empathic and prosocial behavior in autism (Baron-Cohen et al., 2004; Charman et al., 1997; Sigman et al., 1992; Travis et al., 2001).

Nevertheless, it contrasts with previous findings that indicated similar levels of empathic behavior between autistic and non-autistic adolescents during the PEBA paradigm (Zadok et al., 2021). This discrepancy may be caused by the two modifications made to the PEBA paradigm, including the refining of the distress manipulation and conducting the social interaction through the computer. First, the refined and less salient cues of a need-for-comfort included in the current study may have been harder for the autistic adolescents to spot and address. Moreover, by bringing up her difficulties in school in the conversation, in contrast to dwelling on an incoming text message (as in previous administration of the PEBA), the confederate invited the participant to respond, and by that, situated a more demanding social condition. These explanations are supported by previous reports of discrepancies in the performance of autistic individuals, who succeeded in explicit tasks of social reasoning but struggled in complex naturalistic social situations (Klin, 2000; Klin et al., 2003). Meaning, when social information is given more explicitly, some autistic individuals manage to rely on social skills and scripts, to respond in a conventional manner (Livingston et al., 2017).

Second, the administration of the interaction through a video conversation medium and not as a face-to-face interaction may have compromised social engagement and mutual understanding (Sherman et al., 2013). In particular it may have burdened autistic adolescents’ efforts to communicate and converse with the social partner (Howlin, 2004). In contrast, non-autistic adolescents may have felt more comfortable in video-conversation with a stranger, compared to face-to-face “get to know” conversations. Indeed, the increased anonymity and distance in computer-based communication was suggested to paradoxically promote empathy among non-autistic individuals (Jiang et al., 2011).

Importantly, synchronization findings were partially based on automated facial expressions detection. As demonstrated, automated facial expressions analysis produced less interpretable data for dyads involving autistic adolescents compared to those with non-autistic adolescents. These results may suggest a distinct behavioral pattern in front of the Zoom camera, such as the tendency to look away, and/or not look directly at the camera, however they do not offer insights into behavior when participants are not facing the camera. Similarly, an autistic individual looking away may or may not be following the interaction. Another possibility is that the automated facial expression analysis software detected facial expressions, but struggled to interpret them accurately in autistic individuals. This suggests that such software may be biased towards interpreting non-autistic expressions. Future studies should explore whether facial expression analysis software could benefit from adjustments tailored to better capture autistic expressions.

As hypothesized, autistic adolescents were assessed as less synchronized with the social partner throughout the interaction. This adds to existing findings suggesting less behavioral synchrony among autistic individuals when interacting with non-autistic social partners (Fitzpatrick et al., 2016; Kaur et al., 2018; Ochi et al., 2019) and supports previous findings showing less behavioral synchrony among autistic adolescents during the PEBA interaction (Zadok et al., 2021). Yet in contrast to our hypothesis, adolescents in both groups displayed similar patterns of change between the interaction stages. As such, behavioral synchrony increased from the discouragement stage to the return to friendly stage. The increase in behavioral synchrony in the return to friendly stage may be related to a feeling of relief due to being able to retain a casual, friendly conversation. Such an increase in the final stage of the interaction may also reflect a sense of ease in the conversation, as the interaction progressed. This pattern of change in behavioral synchrony characterized both non-autistic and autistic adolescents. This contrasts with previous findings, which indicated a decrease in behavioral synchrony in the face of the partner’s distress among non-autistic adolescents, whereas autistic adolescents displayed constant (and lower) levels of synchrony (Zadok et al., 2021). This gap may have resulted from the different ways in which behavioral synchrony scores were computed in each study. In the current study, eye gaze synchrony and body posture synchrony were not coded, because of the difficulty to reliably evaluate such behaviors in video conversations. Missing these behaviors in the social communication picture may have blunted differences in behavioral synchrony dynamics between autistic and non-autistic adolescents. Meaning, it is possible that overlooking aspects of synchrony that are related to eye gaze, and body posture and movement, concealed subtle changes in behavioral synchrony dynamics of non-autistic adolescents, and yielded a more constant display of behavior, which is like the behavior of autistic adolescents previously observed. Moreover, automated facial expression analysis revealed a trend of less synchrony in dyads with autistic adolescents compared to dyads with non-autistic adolescents. This finding adds to the existing body of evidence regarding facial expression synchrony in autism (Trevisan et al., 2018) and provides a demonstration of it during semi-naturalistic interactions. Considering the role of facial expression synchrony in mentalizing and empathy, it can be suggested that the low levels of synchrony in interactions with autistic adolescents compromised their empathic behavior. The role of facial expression synchrony in forming a shared emotional experience may be especially important when interacting through video, where other social signals, such as body language and eye contact, are somewhat hindered. Alternatively, the reduced empathy may have hampered facial expression synchrony, as these two emerge in an interaction and facilitate each other bidirectionally (Shamay-Tsoory et al., 2019). As such, Halberstadt and Niedenthal (2001) have shown that knowledge about the social situation and an understanding of it affects early stages of processing of facial expressions, including synchronizing with them. It is possible that autistic adolescents experienced the social situation as ambiguous and confusing, and thus, coordination with the social partner was hampered.

Another possible explanation to the lower levels of joyful expression synchrony in dyads with autistic adolescents may be that autistic adolescents smiled less during the interaction overall. To examine this option, group differences in smiling frequency were examined. Results indicated lower frequency of smiling among autistic participants, compared to non-autistic participants (*t*_*w*_(38.41) = 2.5, *p* = 0.02). This strengthens the possibility that the differences in facial expression synchrony found were at least partly driven by lower occurrence of smiles in the autistic group. However, it may be argued that these low frequencies are driven by less synchronous behavior. In other words, if a participant tends not to synchronize their facial expression with the friendly confederate, they may show fewer smiles overall. To clarify these dynamics, a differentiation between initiative smiles versus reactive smiles should be examined in future studies. Such an examination could extend our understanding of behavioral synchrony and its underlying mechanisms among autistic individuals. Importantly, the automated facial expression analysis focused on an averaged measure of synchronization in time lags that considered both the confederate and the participant as followers, i.e., as matching the other’s facial expression. Thus, the lower synchrony found in dyads with autistic adolescents may indicate a reciprocal breach in the interaction, where both partners fail to synchronize their facial expressions with each other. This relatively low facial expression synchrony can be considered to result from the dissimilarity between the two partners interacting, considering their manners of communicating and sharing information and emotions. This concept of breakdown in mutual understanding, described in recent years as ‘the double empathy problem’ (Milton, 2012), offers a different framing to our findings. Accordingly, considering the mutual breach in interaction may be crucially important to the understanding of autistic individuals’ social experiences (Milton et al., 2022). For example, autistic adults report experiences of being misunderstood and of unacceptance, as well as negative consequences of these experiences on their well-being (Camus et al., 2022). The causes of such experiences may be better understood by examining ‘cross neurotype’ social interactions (Milton et al., 2022). Supporting that perception, Crompton and colleagues (2020) demonstrated higher rapport and more effective information transfer in interactions of similar partners (i.e., autistic partners or non-autistic partners), compared to interactions of mixed pairs (i.e., an autistic partner with a non-autistic partner). Nevertheless, a few studies investigating behavioral synchrony showed that, like mixed dyads, autistic dyads demonstrated reduced motion synchrony (Georgescu et al., 2020) and reduced alignment in a computer game that required players to generate and align the meaning of non-verbal communicative signs (Wadge et al., 2019). These mixed preliminary findings stress the need for further investigation of behavioral synchrony in the interactions of autistic dyads.

Further alteration between autistic and non-autistic adolescents was found in emotion recognition abilities. As in previous studies showing emotion and mental state recognition difficulties in autistic adults and children (Golan et al., 2006, 2015), autistic adolescents scored lower than non-autistic adolescents in a task that included recognition of complex emotions from videos of facial expressions. Intriguingly, emotion recognition abilities were not associated to any of the behaviors measured during the PEBA interactions. This finding may indicate that emotion recognition difficulties may not (fully) account for the alterations autistic adolescents display in synchronous and empathic behavior. It challenges previous assumptions relating socio-emotional behavior disparities in autism to emotion recognition difficulties (Baron-Cohen et al., 2009; Bölte et al., 2003; Fitzpatrick et al., 2018) and strengthen the notion that automatic affective processes and their dynamics, such as behavioral synchrony patterns, play a central role in such social alterations.

The relations between the study measures and participants’ level of autistic traits (parent reported using the SRS-2) were examined while treating autistic traits as a continuous measure across both autistic and non-autistic participants. Results revealed that the level of autistic traits was negatively related to behavioral synchrony in all three stages. These results support previous findings that synchronous behavior, regardless of context, is associated with the degree of autistic traits (Cheng et al., 2017; Glass & Yuill, 2024). Additionally, the analysis revealed a trend of negative correlations between autistic traits and empathic behavior. These results support previous findings suggesting that a difference in social understanding may lead to an altered empathic, or prosocial behavioral response (Liu et al., 2023) Furthermore, the analysis revealed a negative relation between autistic traits and facial expression synchrony in the friendly stage, implying that facial expression synchrony in neutral contexts may predict the degree of autistic traits. However, these preliminary findings should be considered with care due to the limited sample size. While combining both groups improved statistical power, further investigation with larger and more diverse samples is necessary to clarify the complex relations between autistic traits, empathic behavior, and synchronous behaviors in both neutral and emotionally charged contexts. Additionally, including physiological measurements during the PEBA interaction may extend our behavioral findings and deepen the understanding of the affective aspects of empathic behavior. Finally, since autistic youth tend to experience more anxiety than their non-autistic peers (Vasa et al., 2015), anxiety may play a part in their social interaction and empathic behaviors. Previous studies have demonstrated that individuals with higher levels of anxiety exhibit less behavioral synchrony in communication (Asher et al., 2020; Shatz et al., 2024). Given the frequent co-occurrence of anxiety in autistic individuals, the impact of anxiety on the behavioral synchrony and empathic behavior of autistic adolescents warrants further research. Specifically, future studies should examine whether anxiety might serve as either a moderating or a mediating factor in the relationship between an autism diagnosis and asynchronous social behavior—suggesting that anxiety, rather than merely autism, could be responsible for differences in behavioral synchrony.

Our findings are limited and should be interpreted with caution, as they partially rely on automated facial expression detection. Our findings are further limited by the small sample size of male adolescents, all of whom with intact intellectual functioning. Indeed, a larger sample size may achieve a greater statistical power and may further clarify the effects and trends that were observed in this study. Another limitation of the current study is the reliance on an online video call platform that may have lags in transmission, which can range up to 200ms depending on factors such as the platform used and geographic location (Chang et al., 2021). Measuring and interpreting such lags presents a potential limitation in accurately assessing synchrony in video calls. We addressed this limitation by including the non-autistic control group, which was arguably prone to similar technical issues. Another limitation that should be addressed in future studies is the inclusion of adolescents with varying cognitive abilities, to better represent the diversity of skills and difficulties in autism. In addition, future studies should include females, and may enable an investigation of possible gender differences in synchronous and empathic behaviors. Indeed, accumulating evidence suggest gender differences in autistic social interaction and communication (Wood‐Downie et al., 2021). Moreover, including dyads of the same sex, and not only of the opposite-sex as was done in this study, is warranted to complete a fuller characterization of such gender differences.

We conclude that autistic adolescents and non-autistic adolescents differ in their empathic behaviors in response to the discouragement of a NA conversation partner. As emotion recognition difficulties don’t appear to explain this disparity, the role of behavioral synchrony may be central. Therefore, targeting coordination and alignment with social partners may be beneficial for autistic individuals who seek to better integrate in the social field.
